# Around the Hospitals

**Published:** 1894-07-14

**Authors:** 


### Around the Hospitals.

[Notice to the Managers of Institutions.—Special space is now reserved for the insertion of notices of hospital meetings and festivals. The Editor requests that all notices may reach The Hospital Office, 428, Strand, at least one week before the date of each meeting.]

**The Poplar Hospital for Accidents.**—The Poplar Hospital has been brought prominently into notice recently, when the whole of the building was opened by the Prince and Princess of Wales. During the past year the management have been doing their best to meet the heavy requirements of the work, amidst the inconvenience of temporary arrangements. Though situated in a crowded, poor, and in some respects uninteresting neighbourhood, within the hospital there is an enthusiasm and brightness which dispels the effect of sombre surroundings. Those who work at Poplar are genuinely interested in their labours, and they receive the greatest encouragement and consideration from their committee and popular chairman. The work is very arduous, the number of accidents requiring attention during the day averaging ten per hour. The Donald Currie ward was opened during the past year, named in recognition to the generous friend of the institution, Sir Donald Currie. At the annual dinner of the hospital Sir Donald Currie gave £500 towards the completion of the new building, he knowing from personal experience the useful work done. The policy at Poplar is against incurring debt. The increase of patients will tax the energy of the chairman and all good friends of the hospital to secure an adequate income. The working men and the poor generally in Poplar do a great deal for their hospital, but there are no rich inhabitants to turn to, and it is to those from wealthier quarters, who sympathise with the risks and sufferings of dock workers, that the hospital has to look for the larger amount of its funds. In spite of the fact that for one month during 1893 the hospital was entirely closed, and for another two wards were closed, the patients, amounting to 443, were under 200 less than during the previous year. The out-patients number 9,755. The financial year ended with a credit balance of upwards of £800.

**The Alexandra Hospital for Children with Hip Disease.**—This hospital contains 68 beds for the treatment of a disease so common amongst the poor, the sufferers from which do not readily gain admission into general hospitals owing to the lengthy nature of the complaint. The treatment is not entirely free, 4s. 2d. being required as a weekly payment for each little patient. Although a small charge is made, the expenses of the hospital, necessarily heavy ones, are never provided for by the available income; also funds are required for rebuilding, as the present structure is not all that could be wished. The convalescent home in connection with the institution, at Bournemouth, is now abolished, but owing to the kindness of a good friend of the hospital, Miss Weymss, another has been opened for eight patients at Painswick, near Stroud. During the past year 144 little patients have been benefited. In the out-patient department 119 children have received treatment. The management do their best to render the lives of the little inmates bright, and visitors who come for the purpose of amusing and instructing the children are welcomed in the wards. We notice one feature in the report of the Alexandra Hospital which we do not recollect to have seen elsewhere, and that is a series of letters, in commendation of the institution, published in its pages. The Alexandra Hospital stands in no need of such testimonials, and the good effected by it on behalf of so many suffering children is, we should hope, when brought before the public, all that is required to induce them to contribute towards its support.

